# Learning, neural plasticity and sensitive periods: implications for language acquisition, music training and transfer across the lifespan

**DOI:** 10.3389/fnsys.2013.00090

**Published:** 2013-11-20

**Authors:** Erin J. White, Stefanie A. Hutka, Lynne J. Williams, Sylvain Moreno

**Affiliations:** ^1^Rotman Research Institute, BaycrestToronto, ON, Canada; ^2^Department of Psychology, University of Toronto, TorontoON, Canada; ^3^Centre for Brain Fitness, Baycrest, TorontoON, Canada

**Keywords:** sensitive period, learning, plasticity, language, second language, music, transfer, attention

## Abstract

Sensitive periods in human development have often been proposed to explain age-related differences in the attainment of a number of skills, such as a second language (L2) and musical expertise. It is difficult to reconcile the negative consequence this traditional view entails for learning after a sensitive period with our current understanding of the brain’s ability for experience-dependent plasticity across the lifespan. What is needed is a better understanding of the mechanisms underlying auditory learning and plasticity at different points in development. Drawing on research in language development and music training, this review examines not only *what* we learn and *when* we learn it, but also *how* learning occurs at different ages. First, we discuss differences in the mechanism of learning and plasticity during and after a sensitive period by examining how language exposure versus training forms language-specific phonetic representations in infants and adult L2 learners, respectively. Second, we examine the impact of musical training that begins at different ages on behavioral and neural indices of auditory and motor processing as well as sensorimotor integration. Third, we examine the extent to which childhood training in one auditory domain can enhance processing in another domain via the transfer of learning between shared neuro-cognitive systems. Specifically, we review evidence for a potential bi-directional transfer of skills between music and language by examining how speaking a tonal language may enhance music processing and, conversely, how early music training can enhance language processing. We conclude with a discussion of the role of attention in auditory learning for learning during and after sensitive periods and outline avenues of future research.

## Introduction

The auditory cortex (A1) is shaped by our experience with sounds in our environment. Incoming sounds sum in the auditory nerve response. Yet, from this, the neural networks underlying auditory processing extract the features that segregate auditory objects and extract meaning from the signal (Bregman, 1994; Werner, 2012). Language and music are among the most cognitively complex uses of sound by humans; however humans have the capacity to readily acquire both skills early in life as a result of exposure and interaction with sound environments. A central question of neurobiology and human development is whether this learning is contingent on the developmental timing of exposure, that is, whether there may be sensitive periods in development during which learning and its corresponding neural plasticity occur more readily than at other points.

Sensitive periods are epochs in development where specific experiences have enhanced, long-lasting effects on behavior and the brain (Knudsen, 2004; Penhune, 2011). During these times, there is increased sensitivity to regularities in sensory input that are readily extracted through exposure and interaction with the environment. As such, they are an optimal time for learning (Werker and Tees, 2005). The term “critical period” is often used interchangeably with ‘sensitive period’, although important distinctions exist between them. *Critical periods* posit short and sharply defined windows-of-opportunity during which exposure to environmental input causes irreversible changes in brain function and structure, whereas *sensitive periods* involve gradual shifts in sensitivity to environmental input outside of which learning is still possible (Lamendella, 1977; Oyama, 1979). The broader term “sensitive period” will be used here to refer to periods in development in which experience has unusually strong effects on brain and behavior (Knudsen, 2004) and to underscore the potential for learning and brain plasticity to continue throughout the lifespan. Sensitive periods are thought to underpin the development of a variety of auditory skills, from the basic encoding of acoustic information in the primary A1 (De Villers-Sidani et al., 2007; De Villiers-Sidani et al., 2008) to many higher-order aspects of language (e.g., Johnson and Newport, 1989; Kuhl, 2010) and music processing (e.g., Penhune, 2011).

The goal of this review is to better understand the mechanisms by which learning and plasticity occur both during and after sensitive periods in auditory development. In the following sections we first give an introduction to general mechanisms by drawing on animal models of auditory development and perceptual learning. Next, we examine three issues that are specific to human auditory development: (1) the role of language exposure versus training in initiating the formation of language–specific phonetic representations in infants and adult second language (L2) learners; (2) the outcome of training that begins at different points in development on neural and behavioral correlates of sensorimotor, motor and auditory processing using music as a platform; and (3) the extent to which childhood auditory experiences, be it with music or speech, result in domain-general enhancements in auditory and auditory-attentional processing. We conclude with critical considerations about the role of selective attention during and after sensitive periods and present directions for future research.

## Auditory learning and plasticity during a sensitive period

Although there may be multiple sensitive periods, each guiding different aspects of auditory development, the mechanism by which learning and plasticity occurs is similar. At the beginning of a sensitive period, neural representations are rather broadly tuned to relevant environmental stimuli (Dahmen and King, 2007; Scott et al., 2007). Broad tuning is advantageous because it allows the developing brain to perceive and respond to the features of the sensory environment. Throughout the sensitive period, neural representations become increasingly refined and begin to preferentially respond to frequently encountered features (Scott et al., 2007), thereby allowing for more accurate and efficient processing of salient and frequently encountered information (Kuhl et al., 2008).

Across multiple sensory systems, learning and plasticity during sensitive periods is a “bottom-up” process, characterized by a perceptual narrowing in which perceptual discrimination and underlying neural representations become increasingly selective in their responsiveness to environmental input (Werker and Tees, 1984; Scott et al., 2006, 2007; Kuhl and Rivera-Gaxiola, 2008). It is this initial under-specification of neural systems that is thought to drive the rapid changes that are observed during this time in response to exposure to environmental stimuli (Knudsen, 2004). Within the auditory system, perceptual narrowing during specific sensitive periods in development characterizes how infants learn to group speech sounds into language-specific phonetic categories (Werker and Tees, 1984), process culture-specific musical rhythms (Hannon and Trehub, 2005a,b) and harmonic relationships (Lynch et al., 1990), as well as encode basic auditory features in the primary auditory cortex A1 (Zhang et al., 2002).

Animal models of auditory development have informed our understanding of the time course in which auditory experience becomes represented in the primary A1. In prenatal development, animal models show that spontaneous rhythmic sound pulses create rudimentary tonotopic maps (Lippe, 1994, 1995; Jones et al., 2007). Following birth, these underspecified tonotopic maps enhance their response specificity through exposure to complex sound streams in the environment, which result in the formation of highly organized maps that are dynamically regulated by environmental input (De Villers-Sidani et al., 2007; De Villiers-Sidani et al., 2008; Zhang et al., 2001, 2002). For example, De Villers-Sidani et al. (2007) exposed rat pups to a series of repetitive tones and found abnormal tonotopic map development. That is, in these rats more neurons were devoted to processing the frequencies of the repeated tones, with consequently fewer neurons devoted to processing other tone frequencies, relative to rat pups raised in a normal acoustic environment. Evidence for sensitive periods in audition also comes from studies of disrupted or altered auditory input at different ages (see e.g., Zhang et al., 2002; Chang and Merzenich, 2003; Chang et al., 2005; Takahashi et al., 2006). Zhang et al. (2002) exposed 9 days old rat pups and adult rats to 20 days of pulsed white noise, disrupting the normal temporal patterns of neural discharge that represent specific auditory inputs. At 80 days postnatally, they found degraded tuning curves in A1 in noise-reared rat pups. The tuning curves were broader than in control pups, with multiple peaks in their receptive fields. Moreover, this disordered auditory representation was maintained, with the tonotopic map representing only a two-way distinction between high and low frequency sounds. Adult rats, by contrast, did not show any significant changes to their pre-existing auditory neural representations when exposed to prolonged noise pulses. The effects appear to result from exposure during key, and sometimes very narrow, developmental epochs (De Villers-Sidani et al., 2007; De Villiers-Sidani et al., 2008).

## Auditory learning and plasticity after a sensitive period

In contrast to other sensory systems, the A1 appears to have an extended period of heightened developmental plasticity, with changes in cellular organization and connectivity continuing throughout childhood (for reviews see Kral and Eggermont, 2007; Penhune, 2011). Indeed, the A1 shows considerable changes as a result of perceptual training even into adulthood (Recanzone et al., 1993; Feldman and Brecht, 2005; Polley et al., 2006; for reviews, see Fahle, 2009; Blundon et al., 2011; Chun et al., 2013). However, the conditions that induce plasticity appear to change with age and experience; namely, the bottom-up learning of the sensitive period becomes increasingly influenced and gated by top-down processes (Ahissar et al., 1992; Crist et al., 2001; Fritz et al., 2005, 2007; Polley et al., 2006; Froemke and Martins, 2011). Bottom-up and top-down processes describe the two ends of a continuum that describes the relative weight of external environmental signals versus internal cognitive processes in driving cortical map plasticity. Bottom-up learning is largely a data-driven driven process, whereby exposure to frequently encountered stimulus features refines their corresponding neural representations (Scott et al., 2007). Once rudimentary representations and higher-order categories are formed, they begin modulating sensory feature processing in an increasingly top-down manner (Kral and Eggermont, 2007). Attention also provides top-down input that, with development, increasingly interacts with and shapes bottom-up signals (Jagadeesh, 2006). Although both processes interact throughout development, the close of a sensitive period may be in a shift in the relative reliance on bottom-up versus top-down processing in learning.

For example, Polley et al. (2006) selectively trained two groups of adult rats to make a snout press to either the frequency or the intensity of the same auditory stimuli that varied in both dimensions. If bottom-up processes are primarily responsible for adult cortical plasticity, as in juvenile animals, they hypothesized that mere exposure to frequency and intensity variation would be enough to elicit the same plastic changes in the representation of both frequency and intensity in their respective groups. Yet, electrophysiological recordings revealed functional changes in primary and secondary auditory cortices that were associated with perceptual learning of task-relevant stimulus features and not stimulus general features. In other words, a double-dissociation was observed among the groups, with no change in cortical map representations observed for task-irrelevant features. Different profiles of neural plasticity were observed despite exposure to same auditory stimuli, which was taken as evidence that adult cortical plasticity may be modulated by top-down inputs that signal the importance and relevance of particular stimulus features.

Thus, while cortical maturation results in a progressive decline in capacity for bottom-up processes to induce auditory plasticity, concurrent development of higher-order auditory representations (e.g., categories) and other top-down influences such as attention regulation increasingly compliment bottom-up processes to modulate the residual capacity for adult cortical reorganization (Kral and Eggermont, 2007). Although both processes may interact throughout the lifespan, sensitive periods and age-related changes in the propensity for learning from mere exposure may be associated with a developmental shift in the relative reliance on bottom-up versus top-down processes. Language acquisition provides a good illustration.

## Evidence for a sensitive period in the perception of speech sounds

Language is often taken as a classic example of sensitive periods in neurobiology and human development (Lennenberg, 1967; Hensch, 2004; Knudsen, 2004; Kuhl, 2010). However, not all aspects of language display the same temporally defined windows of opportunity. Vocabulary learning, for example, continues throughout life, though there is rapid growth around 18 months of age (Long, 1990; Kuhl, 2010). In contrast, the degree and timing of neuroplasticity for phonology and syntax are thought to be highly sensitive to the age at which language exposure occurs (Werker and Tees, 2005; Stevens and Neville, 2009). Although issues remain concerning the timing and extent to which sensitive periods may guide phonological development, the general consensus is that a sensitive period exists for phonetic learning (e.g., Kuhl, 2010).

### Early language exposure results in a perceptual shift

Language development during the first year of life is characterized by a shift from language-universal to language-specific phonetic perception (Werker and Tees, 1984, 2002, 2005). At birth, innate perceptual sensitivities allow young infants to categorically perceive and discriminate virtually any speech sound in any language, even those to which they have not been exposed (Eimas et al., 1971; Jusczyk and Luce, 2002). However, between 6 and 12 months of age, infants’ auditory systems begin a dramatic perceptual shift that directs how they respond to speech sounds. During this time, which some view as the sensitive period for phonetic learning (e.g., Kuhl, 2010), exposure to the language(s) used in their environment is thought to guide infants’ formation of language-specific phonetic representations that serve optimal processing of their native language(s) (Kuhl et al., 2003). Following Hebbian principles (neurons that fire together, wire together; Hebb, 1949), this exposure strengthens the neural representations for speech sounds in infants’ native language(s), while neural representations of unused phonetic distinctions weaken (McClelland, 2001). Infants’ progressive reductions in sensitivity to phonetic distinctions that are not used in the language(s) of exposure has been documented for a variety of non-native consonant (Werker et al., 1981; Werker and Tees, 1984), vowel (Polka and Werker, 1994; Bosch and Sebastian-Galles, 2003) and lexical tone (Mattock et al., 2008) contrasts.

However, more recently, research has shown that this phonetic shift also results in perceptual gains, conferring an enhanced sensitivity to frequently encountered, meaningful phonetic distinctions in the native language(s) that facilitates future language learning (Kuhl et al., 2005, 2008). For example, Kuhl et al. (2008) reported that event-related potential (ERP) correlates of phonetic discrimination the mismatch negativity, (MMN; Näätänen et al., 1997) measured at 7.5 months in response to native-language phonetic contrasts were positively correlated with measures of vocabulary and syntactic development up to 2 years later. By contrast, larger MMNs in response to non-native phonetic contrasts were associated with fewer words and less complex sentences 2 years later. The authors suggest that infants’ discrimination of the native and non-native phonetic contrasts reflects important differences in brain development: better discrimination of non-native contrasts reflects an immature developmental stage in which the infant’s auditory system has not yet committed to relevant native-language speech patterns, whereas enhanced native-language discrimination is associated with neural circuits that have already begun specializing to the speech patterns present in the input language. This underscores the importance of language experiences during a sensitive period: the earlier language-specific neural representations of phonetic categories are formed, refined and stabilized, the earlier and more efficiently they can guide other aspects of language learning.

What guides infants’ shift in phonetic perception and the formation of language-specific neural representations? There is evidence that this perceptual shift is dynamically regulated by the statistical distribution of phonetic variation in the language(s) that the infant is exposed to, which suggests that a bottom-up learning mechanism also drives the development of speech perception. In their seminal study, Maye et al. (2002) examined infants’ discrimination of a non-native phonetic contrast after a brief 2-min exposure to speech sounds from a phonetic continuum that displayed one of two frequency distributions: (1) bimodal, where tokens from endpoints of the continuum were presented relatively more often; or (2) unimodal, where tokens from the center of the continuum were presented relatively more often. In the test phase, only the infants exposed to the bimodal frequency distribution could discriminate the phonetic contrast, even though both groups were exposed to the same stimuli. The authors posited that sensitivity to the statistical distribution of speech sounds is one tool that infants use to determine which acoustic variations are more reliable and therefore more informative for differentiating phonetic categories in the language(s) they are learning. A bottom-up, domain-general statistical learning mechanism has been proposed to underpin other aspects of early language development, including the ability to accurately segment words (Saffran et al., 1996) and order them according to syntactical rules (Saffran and Wilson, 2003). Thus, the perceptual re-organization associated with the establishment of language-specific phonemic representations appears to develop in a bottom-up manner.

Work with near-infrared spectroscopy (NIRS) suggests that the developmental shift towards differentiating language-specific phonetic contrasts coincides with changes in the auditory network subserving phonetic processing, in particular the development of left-lateralization (for reviews see Minagawa-Kawai et al., 2008; Obrig et al., 2010). For example, Minagawa-Kawai et al. (2007) presented five groups of infants (aged 3–4, 6–7, 10–11, 13–14 and 25–28 months) with vowel duration contrasts that corresponded to across- or within-phonetic boundary changes in their native language (Japanese). Phonemic-specific responses (i.e., larger cerebral hemodynamic responses for across- compared to within-phonetic category changes) were transiently observed in 6 to 7 month old infants, before stabilizing in infants 12 months and older. After 12 months, phonemic-specific responses also began showing a left-hemisphere dominance, as in adult native speakers. The authors interpret these findings as a developmental shift in the mechanisms used for phonetic discrimination—from more general auditory processing at 6–7 months to more linguistic-specific processing after 12 months.

In sum, language-specific left-dominant phonemic category representations appear to develop in a bottom-up manner as a result of language-specific experience during the first year of life. Once a rudimentary version of phonemic category representations exist, they enter into a feedback relationship that increasingly guide speech perception in a top-down manner (Kral and Eggermont, 2007) and bootstrap further language development (Kuhl et al., 2008). Infants’ period of heightened sensitivity to the distribution of phonetic cues in their language(s) of exposure (i.e., the sensitive period for phonetic learning) may end when the underlying neural representations of phonemic categories reach a finite point of specificity and stability (Kuhl et al., 2008). Although this may be advantageous for processing one’s native language(s), it can have deleterious consequences for processing new stimuli with a different distribution of acoustic features. Such is the case for adult L2 learners.

### Exposure versus training in second language learning after a sensitive period

Examining the process and outcome of L2 learning at different points in development provides a unique perspective into sensitive period effects. In particular, examining L2 acquisition in adult learners allows us to examine the extent to which neural systems that were established for optimal processing of one set of inputs (i.e., a first language; L1) can be later adapted in order to process another set of language inputs (i.e., L2) more effectively. Moreover L2 learning can occur at different ages, in a variety of L1 speakers and through different learning experiences (e.g., implicit learning through exposure vs. explicit training). Consequently, L2 acquisition provides a unique model for examining how experiential and maturational factors interact to facilitate or restrict learning throughout the lifespan.

The most controversial issues in the field of L2 acquisition are the extent to which a learners’ age impacts his/her ultimate L2 attainment level and whether there may be one or more sensitive periods in language development that limit lifelong L2 learning (e.g., Singleton and Ryan, 2004; Birdsong, 2006). Successfully acquiring L2 phonology is highly sensitive to the age at which learning begins (for review, see Piske et al., 2001). For example, Flege et al. (1999b) examined the pronunciation skills of a large sample of native Korean speakers who had arrived in the United States between the ages of 1 and 23 years who, upon arrival, began intensive English L2 learning. Results showed a positive correlation between degree of foreign accent and age of arrival (even after controlling for years of education, length of residence and L1/L2 use). In contrast, the correlation between age of acquisition and performance on a grammaticality judgement task was not significant after controlling for these confounding variables. The authors took this as evidence that age of acquisition may exert a greater impact on L2 pronunciation than on morpho-syntactic skills (c.f., Johnson and Newport, 1989 for a discussion of how L2 morpho-syntax acquisition may also be vulnerable to delays in acquisition). Age of acquisition effects have also been reported for the perception of non-native phonetic contrasts (Flege et al., 1999a).

What causes these age of acquisition effects in successful L2 phonological attainment? Difficulties that late L2 learners experience with L2 perception and production after years of regular L2 exposure has been taken as evidence that successful L2 phonetic learning and its corresponding neural plasticity may not be possible after a sensitive period has ended (e.g., see, Long, 1990; Pallier et al., 1997; Sebastián-Gallés and Soto-Faraco, 1999; Sanders et al., 2008). The close of sensitive period(s) for language development and the resulting decreased capacity for L2 learning with age has been tied to brain maturation (e.g., Lennenberg, 1967; Scovel, 1988; Johnson and Newport, 1989). Maturational declines in synaptic density, decreased levels of brain metabolism (Bates et al., 1992), and increased axon mylination (Pulvermuller and Schumann, 1994) may reduce the potential for successful late L2 acquisition. Alternatively, the act of L1 learning itself may also change the way L2 speech sounds are perceived, thus regulating L2 phonological attainment as a function of the developing L1 phonological system (Flege, 2003). According to this view, age of L2 acquisition predicts discrimination difficulty in so far as older learners tend to have had more L1 experience and thus more opportunity to develop refined and stabilized L1 representations that are neurally committed to L1 processing (Kuhl et al., 2003). These stabilized L1 representations then compete with the formation of L2-specific representations, making L2 learning more difficult (Hernandez et al., 2005). In effect, brain maturation and prior L1 experience likely co-occur and the age-of-acquisition-effect in L2 phonological attainments reflects complex bidirectional interplay of both brain maturation and early language experience (Bates et al., 2002).

Once the L1 phonological system is firmly established, it may act as a perceptual filter that shapes how late L2 learners perceive L2 speech sounds. This can be maladaptive depending on the similarity and degree of acoustic overlap between the L1 and L2 phonetic categories (Flege, 1995a,b; Kuhl and Iverson, 1995; Strange, 2011). The classic example is the persistent difficulty that many native Japanese speakers have with perceiving and producing English /r/ and /l/. This contrast is challenging for many Japanese speakers (particularly those who began learning English later in life) because, unlike English, Japanese groups the phonetic units /r/ and /l/ to one phonemic category (Japanese /r/), thereby treating any acoustic differences between the units as irrelevant (Iverson et al., 2003; Aoyama et al., 2008). For example, Raizada et al. (2010) showed that native English speakers exhibit two distinct patterns of fMRI activity in right Heschl’s gyrus when listening to the English syllables “ra” and “la”, whereas native Japanese speakers tended to exhibit similar activation patterns for each syllable type. Moreover, the degree to which Japanese speakers showed separation between English “ra” and “la” predicted discrimination performance. The tendency for L2 learners to activate the same groups of auditory neurons for processing L1 and L2 speech sounds may explain why non-native phonetic discrimination is so challenging.

Following Hebbian rules (Hebb, 1949), the more neurons within one region fire in response to two different L2 phonemes, the more that pattern is reinforced (see McClelland, 2001 for a discussion). This makes late L2 learning after a sensitive period unlikely to occur through bottom-up processes triggered by exposure alone; that is, neural systems “optimized for performance, may not be optimal for learning” (Thompson-Schill et al., 2009, p. 260). As such, late L2 learners face more difficulties with accurate L2 phonetic perception, which subsequently affects the development of motor programs necessary to produce the subtle difference between L1 and L2 phonemes (Flege, 2003).

Does this mean that it is impossible for successful L2 learning to occur after a sensitive period has closed? Not necessarily. Although delayed L2 exposure may reduce the likelihood of successful learning and plastic changes occurring through exposure alone, many studies have shown that explicit L2 phonetic training can induce both functional changes in brain activity (Callan et al., 2003; Golestani and Zatorre, 2004; Zhang et al., 2009) and successful learning in adult learners (Guion and Pederson, 2007; Kondaurova and Francis, 2010). Phonetic training teaches learners to discriminate L2 speech sounds that not used contrastively in the L1 and are, thus, difficult to differentiate, either because they activate a single L1 phonetic category or are filtered by the L1 phonological system and therefore do not effectively activate any category (Flege, 1995a,b; Kuhl and Iverson, 1995). Explicit training can induce learning by overtly specifying regularities in the signal or by directing learners’ attention to particular forms (DeKeyser, 2003). Such training takes advantage of adults’ propensity for top-down learning, which can allow L1 representations to adapt to the new L2 input (Archila-Suerte et al., 2012).

The method of phonetic training is also important. For example, Guion and Pederson (2007) tested monolingual English speakers on their discrimination of non-native Hindi contrasts before and after being randomly assigned to either a sound- or meaning-attending training group. The sound-attending group was instructed to listen for sounds of Hindi words, while the meaning-attending group was instructed to listen for the meaning of the same words. The sound-attending group showed greater improvement in a categorical discrimination task, particularly for the most difficult contrast.

Training that teaches learners to redistribute their attention to L2 speech sounds may be particularly effective in improving L2 phonetic perception. Kondaurova and Francis (2010) examined the impact of three phonetic training methods on native Spanish speakers’ perception of an English-specific vowel contrast (/i/ versus /I/; as in *sheep* and *ship*) that is not used in Spanish. Native English speakers distinguish these vowels using two acoustic dimensions, spectrum (vowel quality) and vowel duration. Spanish speakers, by contrast, tend to rely predominately on vowel duration, leading to difficulties discriminating the contrasting vowels. Kondaurova and Francis (2010) assigned Spanish speakers to one of three training conditions: vowel spectral enhancement, vowel duration inhibition, or natural correction (which resembled natural language exposure). Results on identification and discrimination tasks showed that while performance for all three groups improved Spanish speakers’ relative use of vowel quality cues, the vowel duration inhibition training was the most effective in reducing reliance on duration cues (although vowel enhancement training was also effective relative to natural correction training).

Several neuro-imaging studies also have reported functional changes in cortical activity during phonetic processing as a result of perceptual training (e.g., Callan et al., 2003; Golestani and Zatorre, 2004; Zhang et al., 2009), suggesting potential for cortical plasticity, even after a sensitive period. For example, Golestani and Zatorre (2004) trained monolingual English speakers to identify Hindi speech sounds as belonging to either dental or retroflex phonetic categories, a phonetic distinction that is not used in English. After only 5 h of training, results showed significant behavioral improvements and functional changes within cortical areas that are used during the classification of native language speech sounds, including within the left superior temporal gyrus (an area associated with phonemic perception; Liebenthal et al., 2005), the left inferior frontal gyrus, and the left caudate nucleus (areas associated with speech articulation; Hickok and Poeppel, 2007). Correlations between degree of success in learning to identify the contrasting phonetic units and changes in neural activity were also observed. These findings underscore how even relatively short periods of phonetic training can induce functional changes in L2 phonetic processing.

Most neural imaging studies of foreign-language phonetic training involve naïve listeners or relatively low proficiency L2 learners participating in short training periods (e.g., ranging from a few hours to a few weeks). Thus, it is unclear the extent to which any behavioral or neural activity differences observed between learners and native speakers also characterize more proficient late L2 learners. More longitudinal training studies are needed to examine the extent to which explicit phonetic training, coupled with frequent and extended L2 use, change L2 phonetic representation and processing in a way that ultimately resembles that of early learners and/or native speakers (for a discussion of how L2 proficiency may impact other aspects of L2 processing, see Steinhauer et al., 2009; White et al., 2012).

Adult cortical plasticity, unlike sensitive period related plasticity, requires a mismatch between the functions of an existing neural network and demands imposed by the environment to generate lasting functional and structural change (Lövdén et al., 2010). Purely bottom-up (implicit) learning mechanisms may not be sufficient for adult learners to change pre-existing L1 phonetic representations in order to better differentiate L2-specific contrasts (Archila-Suerte et al., 2012). By contrast, top-down processes evoked by explicit training that is goal-oriented progressively adapts to participants’ performance, provides feedback and directs attention to the relevant L2 features that require encoding, may enhance post-sensitive period L2 learning by allowing learners to attend to the mismatch between their current and goal-state performance and initiate plastic changes (see Ullman, 2001 for a similar argument about the relative role of declarative and procedural memory in initial stages of L2 syntax acquisition).

## Learning music through training during a sensitive period

Like language, music relies heavily on auditory processing. However, unlike language, music training is a formal process where lessons typically occur early in life, and are quantifiable (Bengtsson et al., 2005; Wan and Schlaug, 2010; Penhune, 2011). This makes musicians an optimal population for studying the effects of sensitive periods on brain and behavior (Steele et al., 2013). Music training also allows us to examine the brain’s capacity to learn and change as a result of training at different ages and examine the processes and skills that are differentially affected by this learning.

Within the last fifteen years, there has been a proliferation of studies examining the neural functioning of adult musicians as compared to non-musicians (e.g., Halpern and Zatorre, 1999; Blood and Zatorre, 2001; Koelsch et al., 2003; Zatorre, 2003). Music training has been associated with volumetric differences in the primary and secondary A1 (Schneider et al., 2002; Bermudez et al., 2009), planum temporale (Schlaug et al., 1995b), corpus callosum (Elbert et al., 1995; Schlaug et al., 1995a; Schmithorst and Wilke, 2002; Sluming et al., 2002; Lee et al., 2003), and motor areas associated with one’s instrument of practice (Amunts et al., 1997; Pantev et al., 1998). Some of these differences have been shown to be functionally relevant. For example, Schneider et al. (2002) found that musicians showed bilateral differences in gray matter volume in anteromedial portion of Heschl’s gyrus that were 130% larger than in non-musicians. This size difference was correlated with melody discrimination performance, such that greater differences were associated with better performance, suggesting that volumetric increases are functionally relevant and enhance music processing abilities.

Collectively, studies examining cognitive and motor performance in musicians versus non-musicians provide a platform from which we can explore the developmental aspect of music training—does music training result in differences in brain structure and function or are there pre-existing structural differences that allow one to excel at music? As the majority of the studies that compared musicians to non-musicians did not report the age at which musicians started their training, they do not allow us to examine whether training that begins early in life is necessary to experience these changes. Of the studies that do report the age at which musicians began their training (Elbert et al., 1995; Schlaug et al., 1995a; Amunts et al., 1997; Sluming et al., 2002; Lee et al., 2003), only a few specifically test for age-related differences in neural structure and function. These studies demonstrate that, as compared to training that begins later in life, early music training is related to enhanced motor processing and representational plasticity (e.g., Elbert et al., 1995; Amunts et al., 1997), greater bimanual motor synchronization (e.g., Schlaug et al., 1995a), and sensorimotor integration (e.g., Watanabe et al., 2007; Steele et al., 2013), suggesting that sensitive periods also may exist in the domain of music acquisition. To facilitate a more nuanced understanding of the relationship between sensitive periods and auditory processing, we will discuss how early versus later music training can affect changes at the motor, sensorimotor, and cognitive levels.

### Sensitive periods in motor processing

Several studies used regression models to examine whether age of starting musical training could account for structural differences in the brain (e.g., see Elbert et al., 1995; Amunts et al., 1997). Elbert et al. (1995) examined string players who started musical training across a range of ages (from 5 to 19), and found that the earlier string instrument training began, the more extensive the cortical network responses to tactile stimulation. Similarly, Amunts et al. (1997) found that the age at which keyboard players began their music training was negatively correlated with the size of the intrasulcal length of the precentral gyrus. Together, these findings suggest that the motor cortex can exhibit long-lasting structural adaptations that are induced by specific experience. The specificity of these effects are a function of the kind of experience musicians have with their instruments, which suggests that age of onset of training plays an important role in driving the structural and functional changes seen in adult musicians.

Bimanual motor performance also may be impacted by the age at which music training begins. In one of the earliest studies to directly test the effects of age of commencement of music training on neural structure, Schlaug et al. (1995a) found that the mid-saggital anterior corpus callosum (maCC) was significantly larger in musicians who started music training before age 7 versus musicians who commenced training after that age. Moreover, the maCC in both musician groups was significantly larger relative to a control group of non-musicians. Similarly, Lee et al. (2003) found further evidence for a link between early commencement of music lessons (i.e., before age seven) and increased maCC size, which was related to continuous practice of bimanual motor training.^1^

Further support for a sensitive period for bimanual performance comes from studies on the plasticity of the maCC. The maCC undergoes significant structural and functional changes between ages six to eight. These changes, in turn, may affect the possible degree of cortical plasticity and the extent to which training after this age results in the same degree of cortical reorganization (Chiang et al., 2009; Westerhausen et al., 2011; Kurth et al., 2012).

### Sensitive periods in sensorimotor processing

Early music training may also impact sensorimotor integration, both neurally and behaviorally. Steele et al. (2013) tested if music training might have a differential impact on plasticity in white-matter fibers connecting sensory and motor regions, resulting in better sensorimotor integration. Using diffusion tensor imaging they found that early-trained musicians had greater connectivity in the posterior midbody/isthmus of the corpus callosum. Fractional anisotropy in this region was related to age of onset of training and sensorimotor synchronization of performance. From this, the authors posited that training before age seven results in changes in white-matter connectivity and that these changes “may serve as the scaffold upon which ongoing experience can build” (p. 1282).

Behaviorally, Watanabe et al. (2007) compared adult musicians who began music instruction early (before age 7) and late (after age 7) though they were matched for years of experience and amount of current practice. Participants were tested on their ability to tap in synchrony to a visually presented complex rhythm. Results showed that even though both groups had experienced many years of music training, the early training group showed better synchronization with music rhythms compared to the late training group. This suggests that early training may impact neural systems involved in sensorimotor integration and timing to a greater extent than later training. Likewise, Bailey and Penhune (2012) reported similar results on an auditory rhythm synchronization task, which was taken as evidence that there may be sensitive periods during which music training has long-lasting impacts on rhythm synchronization and other musical skills.

However, important considerations must be kept in mind when interpreting the results of cross-sectional studies (i.e., the studies on music training discussed thus far) and the conclusions they make about sensitive periods. Importantly, cross-sectional studies do not allow us to investigate the causality of differences between musicians and non-musicians. Differential innate predispositions for musical ability may confound these studies and could explain differences between those who began music training earlier independently from the brain’s capacity to learn and change as a result of age of training onset. Additionally, musicians with early-onset training typically have more training than those who began later (see Watanabe et al., 2007; Bailey and Penhune, 2012) or are younger at the time of testing. Both of these factors could account for differences in brain structure and function and in behavioral performance. Finally, cross-sectional studies involve retrospective evaluation of the extent to which the nature, quantity and quality of training were similar across all participants and therefore interpretations of a musical advantage may be somewhat unreliable.

The first longitudinal study to examine structural brain and behavioral changes in the developing brain as a result of music training was conducted by Hyde et al. (2009). They investigated whether 15 months of instrumental music training in 6-year-old children would provide benefits beyond participation in weekly school-based group music classes. Hyde et al. (2009) searched the brain for local brain size differences between groups and found no behavioral or brain differences between the two groups of children at baseline. After 15 months, the children in the instrumental training group showed greater improvements on finger motor tasks and melody/rhythmic tasks post-test, but, importantly, not on the non-musical tests. The instrumental training group also demonstrated greater relative voxel size change as compared to controls in motor regions (e.g., precentral gyrus), corpus callosum, and Heschl’s gyrus. These findings are important because they suggest that the neuroanatomical differences seen in adult musicians relative to non-musicians may result from intensive music training rather than a biological predisposition to music (Norton et al., 2005; Schlaug et al., 2005). Moreover, Hyde et al. (2009) illustrate several key points: (1) early music training may indeed *lead* to substantial neural changes that were not apparent at the start of training, and are thus not due to pre-existing differences in brain structure; (2) the type of music training received may be an important factor in determining the degree and kind of structural changes observed in the brain; and (3) benefits conferred from music training can manifest in a relatively short time (15 months) in young children.

### Effects of early musical training on auditory processing

In addition to providing evidence that musicians exhibit enhanced motor and sensorimotor processing relative to non-musicians, there are also a number of studies that demonstrate that early music training can impact multiple levels of auditory processing. For example, Pantev et al. (1998) measured the cortical representations of highly-skilled musicians using functional magnetic source imaging (single dipole model). The age-of-onset of musical training ranged from three to twelve. Dipole moments for piano tones, but not for pure tones of similar frequency, were enlarged by approximately 25% in the musician group, relative to the non-musician controls. Enlargement was inversely correlated with the age at which musicians started to practice, such that the younger the musicians were when they started to practice, the larger was the cortical reorganization in response to piano tones. Pantev et al. (1998) suggested that use-dependent functional reorganization extends across the sensory cortices, reflecting the pattern of sensory input processed by the participant as his/her musical skills develop.^2^

Similarly, Shahin et al. (2004) measured auditory evoked potentials (AEPs) elicited by piano, violin, and pure tones in four- and five-year-old children enrolled in Suzuki music lessons and non-musician controls. AEPs reflect the development of mature synaptic connections in the upper neocortical laminae that occurs between ages 4 and 15. Results showed that music training affected the AEPs at multiple stages of auditory processing. Compared to controls, Suzuki students exhibited larger P1 and P2 components when listening to their instrument of practice (piano or violin). Moreover, the AEPs observed for piano tones in the music students were comparable to those found in non-musician children three years older. This suggests that musical training can influence and expedite the shaping of neural development.

This neural development, especially the sub-cortical auditory plasticity seen in young musicians, can persist into adulthood (Skoe and Kraus, 2012). Skoe and Kraus (2012) showed that adults who received formal music lessons as children (but who had not played in many years) had more robust brainstem responses to sound than those who had never received lessons. Neural response quality increased significantly from those who had no music lessons during childhood, to those who had 1 to 5 years, to those who had 6 to 11 years. Similarly, Zendel and Alain (2012) found that this benefit may persist into old age. They compared older amateur and professional musicians who started music lessons before age 16, all of which had continued to play throughout their life. They found ongoing music playing mitigated the central auditory processing declines typically associated with aging. Collectively, these findings clearly demonstrate that early training affects the brain, leading to life-long changes in brain function.

### Pitch memory and absolute pitch

Although age of start of musical training is not generally the focus of most studies examining the cognitive benefits of musical training, absolute pitch (AP) is an exception. AP is the ability to identify or produce a specific pitch without a reference pitch (Baggaley, 1974). Levitin (1994) proposed a two-component theory of AP, which posits that AP is comprised of pitch memory and pitch labeling.

Pitch memory is the ability to maintain and access stable, long-term representations of specific pitches in memory (Levitin, 1994). It is a common ability found in both musicians and non-musicians, as a result of everyday exposure to music (Terhardt and Ward, 1982; Terhardt and Seewann, 1983; Halpern, 1989). For example, Levitin (1994) investigated pitch memory in participants with and without musical training. When instructed to sing several bars of their two favorite songs, both groups came within two semitones of the original recordings for both songs, suggesting that everyone—musicians and non-musicians alike—posses pitch memory ability. In pursuit of a related question, Schellenberg and Trehub (2003) had non-musician adults hear a version of a familiar TV theme song played at the standard key and transposed by either one or two semitones. The participants identified above chance which excerpt was in its original key. Similar findings have been observed in children (9 to 12-year-olds; Schellenberg and Trehub, 2008) and infants (Volkova et al., 2006), who were also able to recognize the correct key of familiar recordings, suggesting that pitch memory develops early in life.

Trehub et al. (2008) indirectly addressed whether or not a sensitive period exists for AP by studying the effects of age and culture on children’s memory for the pitch level of familiar music. English speaking Canadian nine- and ten-year-olds were able to distinguish between the original pitch level of familiar television theme songs and foils that were pitch-shifted by one semitone, whereas five- to eight-year-olds could not make this distinction. Conversely, Japanese five- and six year-olds could distinguish the pitch-shifted foils from the originals, performing significantly better than their same-age Canadian counterparts. Trehub et al. (2008) suggested that these differences may stem from Japanese children’s use of a pitch-accent language rather than a stress-accent language (English), thus affording these children additional experience with musical pitch labels. These findings suggest that language type (e.g., pitch- versus stress-accent language) may determine when pitch memory abilities come online and that increased experience with pitch discrimination, whether through language or increased exposure to music, can improve pitch memory (as in the case of the improvement between the five- and six-year-old Japanese children’s performance). The finding that five- to eight-year old Japanese children performed better than their Canadian age-matched counterparts, and that the Canadian children could not discriminate the pitch change until age nine and ten, suggests that experience with a pitch-accent language bootstraps pitch memory abilities earlier than experience with a stress-accent language.

Pitch labeling—the rare ability to attach a meaningful label, such as D#, A440, or Do, to pitches—is the hallmark of AP (Levitin, 1994). Because it requires knowledge of note names, its prevalence is restricted to those with music training (Schellenberg and Trehub, 2008). The probability of developing pitch labeling, and thus AP, substantially increases if music training begins prior to age 6 to 7 (Sergeant, 1969; Miyazaki, 1988; Baharloo et al., 1998; Gregersen et al., 1999; Brown et al., 2002; Deutsch et al., 2006; Miyazaki and Ogawa, 2006), suggesting that AP shows signs of having a sensitive period (Bachem, 1940; Sergeant, 1969; Miyazaki, 1988; Gregersen et al., 1999; Russo et al., 2003; Levitin and Rogers, 2005; Deutsch et al., 2009; Lee et al., 2011). For example, Schellenberg and Trehub (2008) found that early music training is the best predictor of pitch labeling. However, it is unclear whether these age-effects reflect some of the confounding factors that are related to age or maturational differences in the brain’s capacity to reorganize its cortical representations of pitch as a result of music training at different ages.

## Transfer of auditory skills between music and language

Like language, music appears to have sensitive periods. Although neural network differences exist between music and language (Zatorre et al., 2002), they both rely on many similar sensory and cognitive processes. They use the same acoustic cues (pitch, timing and timbre) to convey meaning, rely on systematic sound-symbol representations, and require analytic listening, selective attention, auditory memory, and the ability to integrate discrete units of information into a coherent and meaningful percept (Kraus and Chandrasekaran, 2010; Patel, 2011). This overlap in neuro-cognitive systems leads to the possibility that experience or training in one domain may enhance processing in the other (Patel, 2008; for a longer discussion, see Moreno, 2009).

Transfer between music and language is typically studied in the context of how childhood music training impacts language development (for reviews see Moreno, 2009; Strait and Kraus, 2011). In addition, there is new evidence that suggests language experience also may enhance music processing (Deutsch et al., 2006, 2009; Bidelman et al., 2013). Research into music-language transfer provides a unique perspective into sensitive periods effects because it allows us to examine the extent to which early auditory experiences, be it with language or music, alter the functionality of sensory and cognitive systems in a domain-general way.

### The case of language to music transfer

Although the increased prevalence of AP among certain Asian populations has been suggested to reflect genetic factors (Zatorre, 2003), it may also be related to their experience speaking a tonal language. For example, Mandarin and Cantonese use tone (i.e., pitch fluctuations, Deutsch et al., 2004, but see Burnham et al., 2004; Trainor, 2005) to express word meaning. Bidelman et al. (2013) compared adult Cantonese-speaking non-musicians, English-speaking non-musicians and English speaking trained musicians on music-processing tasks (e.g., pitch discrimination and memory). They found that Cantonese speakers’ performance was comparable to that of musicians and enhanced relative to the English speaking non-musicians. Moreover, in a sample of native Mandarin and English speakers attending music schools in their respective countries, Deutsch et al. (2006) found that Mandarin speakers showed a higher incidence of AP than English speakers (but see Baharloo et al., 1998; Gregersen et al., 1999; Baharloo et al., 2000, for a discussion of AP and genetic influences). The greatest incidence of AP was in children who began music training before 8 years of age, regardless of their language background. However, only a small percentage of Mandarin speakers (and none of the English speakers) developed AP if music training began later, suggesting that previous experience with a tone language may gate the closure of a potential sensitive period.

### The case of music to language transfer

Several studies have examined the transfer of skills from music to language. This transfer can be observed at multiple levels (Bidelman et al., 2013; Moreno and Bidelman, 2013), from perceptual (e.g., acoustic parameters, Chartrand and Belin, 2006; Bidelman et al., 2009, 2011; Bidelman and Krishnan, 2010), to cognitive (Anvari et al., 2002; Franklin et al., 2008; Moreno et al., 2009; Chobert et al., 2012; Francois et al., 2012; Marie et al., 2012), to domain-general (e.g., attention and inhibition, Bialystok and DePape, 2009; Moreno et al., 2011a). This work suggests that there may be an association between childhood music training and improved language processing for a variety of language skills, including pitch discrimination in speech (Moreno and Besson, 2006; Moreno et al., 2009), perception and neural encoding of speech in noise (Strait et al., 2009; Strait and Kraus, 2011), and a variety of reading-related measures, including phonological awareness (Bolduc, 2009; Tsang and Conrad, 2011), naming speed (Herrera et al., 2011), the ability to match visual symbols to words, (Moreno et al., 2009, 2011b), spelling (Overy, 2003), vocabulary (Moreno et al., 2011a), and reading comprehension (Corrigall and Trainor, 2011). Moreover, relationships between early music training, enhanced language processing and increased attentional control (Moreno et al., 2011a; Strait et al., 2012) and auditory working memory (Strait et al., 2012) have been observed in children. The collective importance of these findings is underscored by studies that reported associations between childrens’ music training and increased Intelligence quotient (IQ; Schellenberg, 2006) and school performance (Wetter et al., 2009). Furthermore, the enhancements seen in language domains have been shown to correlate with length and intensity of musical training (e.g., enhanced subcortical auditory and audiovisual processing, Musacchia et al., 2007; subcortical processing of vocal expressions of emotion, Strait et al., 2009). These findings have also been demonstrated in music intervention studies (Besson et al., 2011; Bhide et al., 2013; Thomson et al., 2013). For example, Chobert et al. (2012) found that 12 months of active music training enhanced pre-attentive processing of syllabic duration and voice onset time in 8 to 10 year-olds.

Most studies to date have investigated the impact of music training on developing language and cognitive skills in children. Thus, the extent to which similar transfer effects might occur at different points in development is unclear. Whereas training in adults and older children modifies existing neural circuits, in young children it may still influence the initial formation of those circuits. Consequently, training could result in quantitatively and qualitatively different changes, depending on the brain maturation and an individual’s relative position on his/her language development trajectory (for a discussion see Jolles and Crone, 2012). For example, one might predict that music training may have a greater impact on emerging literacy and selective attention skills in younger children because the room for improvement is larger.

### Mechanisms of transfer

Examining the mechanisms by which training may enhance children’s language and cognitive skills can enhance our understanding of how early auditory experiences shape auditory processing. This is important both practically and theoretically. Practically speaking, it is important for developing effective educational programs that maximize the potential for high-quality learning outcomes. Theoretically, it is tied to fundamental questions about the processes by which the brain generalizes and transfers learning from one domain to another (Gazzaniga, 2008). We suggest that transfer between music and language could occur via shared processing in both auditory and attention control systems (Kraus and Chandrasekaran, 2010; Patel, 2011; Strait and Kraus, 2011).

A neurocognitive model that has been used to illustrate music-to-language transfer is Patel (2011) OPERA hypothesis. The OPERA hypothesis details how musical training facilitates recruitment of neural areas that are used in both music and language, such as Broca’s Area (i.e., Overlap) through a learning process that involves precision (P), emotional-engagement (E), repetition (R), and attentional focus (A). The components of the model contribute to increased neural processing precision for all salient acoustic information, whether musical, linguistic, or other. A central proposition of the OPERA hypothesis is that transfer occurs because the basic encoding of acoustic features in speech and music rely on largely overlapping subcortical and cortical networks. Music-to-language transfer occurs because music processing requires acoustic features to be encoded with a higher degree of precision than is typically required when processing speech. High-precision training of particular acoustic features (e.g., frequency, duration) in music that rely on overlapping neural systems in speech, leads to enhanced precision of those features in both domains. This enhanced precision of acoustic features can then feed-forward to influence higher levels of language processing (e.g., phonemic categorization, phonological-lexical processing; Besson et al., 2011). Similarly, experience with particular acoustic features in language (e.g., lexical tone) may facilitate the neural encoding and processing of those same features in music. This potential bidirectionality of transfer between music and language was supported by Bidelman et al. (2013) who found that adult Cantonese-speaking non-musicians’ performance on music-processing tasks was comparable to that of musicians and enhanced relative to English speaking non-musicians.

Patel (2011) hypothesizes that transfer is possible via shared underlying neural networks mediated by enhanced attentional control. The mechanism of these processes may again lie in Hebbian principles (Hebb, 1949), such that stimulation in one network stimulates the complementary domain by nature of overlapping neural networks. The demands of music training reinforce the auditory and attentional networks which, in turn, transfer to other domains (e.g., language) and improve cognitive skills. Specifically, under OPERA, early music training promotes language development by allowing learners to allocate more attentional resources to shared auditory features, thereby enhancing processing of those features as well as the executive control systems that guide auditory attention and inhibition more generally (Kraus and Chandrasekaran, 2010; Patel, 2011; Strait and Kraus, 2011; Moreno et al., 2011a).

**Figure 1:**
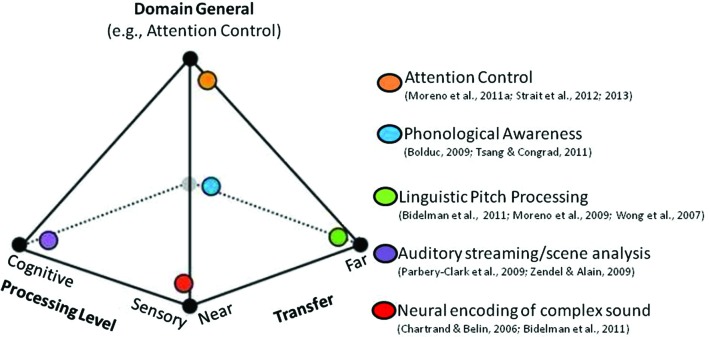
**Music training and transfer effects conceptualized as a multidimensional continuum.** The extent of a transfer effect from one activity to another can be characterized by two continuous, orthogonal dimensions: (1) the level of affected processing (low-level sensory vs. high-level cognitive); and (2) the “distance” of transfer from the domain of training (near vs. far). These complementary dimensions explain a wide range of transfer and cognitive benefits observed across many studies that have examined music-related plasticity (denoted by the colored orbs). The specific amount of benefit and the extent of transfer from music to language (represented by the location within the pyramid model) might be mediated by the extent to which cognitive skills (e.g., attention/inhibition control) are tuned by music training itself.

A second neurocognitive model that builds on the OPERA hypothesis has recently been proposed to explain music-language transfer effects (Moreno and Bidelman, 2013). According to this model, the degree to which transfer occurs and the neural systems affected can be conceptualized as a spectrum along two orthogonal dimensions: Sensory-Cognitive and Near-Far (Figure 1). The *Sensory-Cognitive* dimension characterizes the processing level affected and ranges from low-level sensory processing that is specific to the auditory domain, to high-level domain-general cognitive processes that support language and executive function (e.g., mechanisms that regulate, control and manage attention, working memory and planning). It is supported by research that shows benefits of music training at sensory levels (e.g., experience-dependent plasticity in brainstem AEPs, Kraus et al., 2009; Krishnan and Gandour, 2009; Krishnan et al., 2012) as well as cognitive levels (e.g., music training impacting cortical plasticity, e.g., Münte et al., 2002; Trainor et al., 2003; Zatorre, 2005; Moreno et al., 2011a; Herholz and Zatorre, 2012, and attention/inhibition control, e.g., Moreno et al., 2011a; Strait et al., 2012). The *Near-Far* dimension characterizes the “distance” of transfer (i.e., the degree of similarity) from the domain and context of training to the skills assessed. Examples of near transfer include findings that repeated exposure to the manipulation of auditory patterns leads to the subsequent development of analytic listening skills required for robust auditory stream segregation (Zendel and Alain, 2009), complex sound manipulation (e.g., musical transposition, Foster and Zatorre, 2010), and “cocktail party listening” (Parbery-Clark et al., 2009; Bidelman and Krishnan, 2010; as discussed in Moreno and Bidelman, 2013). Examples of far transfer include when the auditory precision demanded by music training benefits auditory sensory encoding in unrelated domains such as speech and language (Wong et al., 2007; Moreno, 2009; Schlaug et al., 2010; Bidelman et al., 2011, 2013). According to this model, the amount of benefit (i.e., the extent of transfer and the processing levels affected) depends on the length and intensity of training and the degree to which training tunes general cognitive skills. This leaves open the possibility that the particular focus of a given training programs and individual differences in attention control may differentially impact transfer outcomes.

Many studies demonstrate an effect of music training on both language and attentional control (see Kraus and Chandrasekaran, 2010; Strait and Kraus, 2011; Moreno and Bidelman, 2013). For example, Strait et al. (2012) compared the ability to encode speech in noise in children (ranging in age from 7 to 13) who had been receiving regular music training starting before the age of 5 versus those who had not received regular music instruction. The children who had received music training showed enhanced perception of sentences and greater brainstem response to speech sounds in noise. Moreover, this more accurate sentence perception in noise and more robust and faster brainstem encoding of key features of speech sounds were correlated with improved performance on measures of auditory attention. Thus, music training appears to improve the ability to rapidly detect, sequence and encode sound patterns that are deemed important, while suppressing and disregarding irrelevant and meaningless information (Kraus and Chandrasekaran, 2010). These abilities are arguably related to fine-tuning of executive control mechanisms in the brain and, specifically, selective attention mechanisms. Difficulty identifying speech sounds in noise has been argued to be a fundamental deficit for children with specific language impairment (Ziegler et al., 2005) and developmental dyslexia (Ziegler et al., 2009), raising the possibility that music training may provide a benefit for children who struggle with language (Kraus and Chandrasekaran, 2010).^3^

However, many studies of music-to-language transfer employ cross-sectional designs that compare children who have or have not received music training, making it difficult to determine the extent to which differences in language processing reflect the effect of music training *per se* as opposed to pre-existing, innate capacities, motivation, parental involvement or other environmental factors (e.g., Penhune, 2011). To this end, longitudinal studies that randomly assign participants to music or other related training programs are important for understanding the mechanisms of transfer and the extent to which transfer may be sensitive period dependent. In a series of longitudinal studies, Moreno et al. (2009, 2011a,b) and Moreno and Besson (2006) examined the benefit of music training on multiple aspects of language processing by randomly assigning children to teacher-led, computer-based music listening or visual art training programs. For example, Moreno et al. (2009) found that eight year old children showed improvements in EEG correlates of pitch processing in speech after participating in six months of music training as compared to matched children who participated in visual art training (see also Moreno and Besson, 2006). Enhanced auditory processing of important acoustic features in speech may be particularly beneficial for speech perception under challenging listening conditions, as suggested by a musician advantage in detecting speech in background noise (Strait et al., 2012). Moreover, using an intensive (20 day) version of these training programs with younger children (age 4–6), Moreno et al. (2011a) found that music training led to significant enhancements in verbal intelligence (as measured by the Wechler Preschool and Primary Scale of Intelligence –Third Edition, WPPSI-III), with over 90% of the children showing improvements. Significant changes to ERP indices of executive function in a visual Go/No-Go task were also observed, which positively correlated with improvements in verbal intelligence.^4^ Crucially, neither verbal memory nor executive function were significantly enhanced in the control group of children who were randomly assigned to a visual art training group. Collectively, these findings provide causal evidence for the role of music training in enhancing children’s developing language skills. They suggest that children’s language performance may benefit from music training via two sources of transfer: the near transfer of skills within the auditory domain that enhance the encoding of speech and the far/broad transfer of skills between high-level

### Sensitive periods for music-language transfer

Empirical evidence supports that some aspects of language and music are sensitive-period dependent. Given the bidirectionality of the transfer between music and language (i.e., Bidelman et al., 2013), we suggest that there may also be a sensitive period in transfer, such that the effects of training may be greatest during the overlap of the sensitive periods. We also believe that transfer is influenced by the interaction between genetics and environment (i.e., “nature” and “nurture”). AP is an example of this phenomenon. Genetic predispositions have been cited as a contributing factor to AP-development (Baharloo et al., 2000; Drayna et al., 2001; Zatorre, 2003), conferring a general aptitude for frequency encoding. Yet, environmental influences are also important. For example, Schellenberg and Trehub (2008) found that early music training is the best predictor of pitch labeling. However, music training may not be the only “nurturing” auditory experience that contributes to pitch labeling skill. Speaking a tone language is also associated with higher rates of AP (Gregersen et al., 1999; Deutsch et al., 2006), suggesting that tone language experience may bootstrap the ability to meaningfully label sounds, as discussed in relation to pitch memory. Thus, AP appears to be a combination of “nature” and “nurture”, such that some individuals may be born with a pre-disposing genetic disposition that may be more likely to develop into AP when music training and particular language experience is provided early in development. Cross-domain bootstrapping is one of many examples of transfer in and between the domains of language and music.

## Discussion: mechanism of auditory learning and transfer during and after a sensitive period

We suggest that auditory learning and plasticity is possible both during and after a sensitive period; however they differ in their relative reliance on two underlying mechanisms. The difference can be best considered as end points of a continuum between bottom-up and top-down processing mediated by attention (e.g., Strait et al., 2010). During a sensitive period learning is largely a bottom-up process that is triggered by exposure to auditory input. It is an optimal period for learning because underlying neural circuits have not yet been fully specified and are extremely sensitive to input received. Learning occurs through a process of perceptual narrowing that hones in on frequently occurring, and thus important, features in the input (Scott et al., 2007). This occurs gradually as input progressively directs the refinement and stabilization of neural circuits, until a threshold level of stability has been attained, thus, corresponding to the gradual closing of the sensitive period for the skills sub-served by those circuits (Kral and Eggermont, 2007; Kuhl et al., 2008).

After a sensitive period, learning is largely a top-down process that depends on attention to enhance the salience of features in order to encode them. It is a process of changing the structure and efficiency of pre-existing circuits to more optimally process a new input source (Knudsen, 2004; Lövdén et al., 2010). In the case of L2 learning this may involve creating a completely new circuit. In the case of music training, this may involve dramatically improving the specificity of circuits that were created through earlier exposure to music. Both may require explicit training that teaches learners how to best direct their attention to relevant information to initiate plasticity. Indeed, animal studies demonstrate that acetylcholine (a neurotransmitter associated with sustained attention; Sarter et al., 2001) plays an important role in adult experience-dependent plasticity (Kilgard and Merzenich, 1998; Mercado et al., 2001). Acetylcholine is thought to gate learning and plasticity by enhancing the processing of relevant sensory stimuli and filtering out irrelevant noise and distracters (Sarter et al., 2001; Seitz and Dinse, 2007). The release of acetylcholine with attention may mark the importance of particular stimulus features by increasing the responsiveness of neurons, increasing the probability of synchronous firing and strengthening of synaptic connections (Jagadeesh, 2006). Therefore, learning, particularly after a sensitive period, appears to be a gated system, through which attention (via acetylcholine) can facilitate or restrict plasticity (Seitz and Dinse, 2007).

Although bottom-up and top-down processes can be considered as ends of a continuum, the difference between learning during and after a sensitive period can be viewed as one of degree rather than kind: age-related shifts in the relative reliance on each process may be a gradual, rather than an all-or-none, shift. Although bottom-up processes may predominate during a sensitive period, auditory learning may also be facilitated by top-down internal mechanisms and external cues that regulate attention. For example, Conboy et al. (2008) showed that individual differences in 8–11 month old infants’ cognitive control is inversely related to their discrimination of non-native phonetic contrasts (see also Lalonde and Werker, 1995). This suggests that even as early as the first year of life, the domain-general ability to ignore irrelevant information and focus on relevant information may promote early stages of language learning (Diamond et al., 1994). Moreover, infant-directed speech and maternal singing are thought to promote phonetic learning by directing arousal and attention to relevant speech cues (Werker et al., 1996; Trehub and Trainor, 1998). However, the protracted development of the prefrontal cortex and its associated executive functions (Gogtay et al., 2004) and the under-specification of higher-order categories (Kral and Eggermont, 2007) may place an upper limit on the extent to which top-down mechanisms mediate learning early in development. Similarly, although top-down processes may predominate after a sensitive period, bottom-up mechanisms (e.g., statistical learning of speech; Saffran et al., 1997) may continue to operate, although the extent to which they induce learning may depend on the level of specification of the existing neural network (Kuhl et al., 2008) and the efficiency with which the existing network processes new environmental input (McClelland, 2001). Thus, both bottom-up and top-down mechanisms influence learning and plasticity during and after a sensitive period, though the relative reliance on each may change across development.

Viewing learning and plasticity during and after sensitive periods as falling along a continuum between bottom-up and top-down processing mechanisms can help us understand why childhood training is so beneficial. Music training, for example, may be associated with such long-lasting benefits in music, language and attention processing because it strengthens emerging top-down processes at a time when bottom-up mechanisms are still available. Indeed, one benefit of music training may be to expedite the developmental trajectory of top-down control over speech processing (Strait et al., 2013). For example, early music training (i.e., before age 6 or 7) has been found to be associated with more precise encoding of speech and enhanced auditory attention– a benefit observed for both adult and child musicians (ages 7 to 13; began lessons before age 6) relative to age-matched non-musicians (Strait et al., 2013). Significant correlations between attention and neural encoding of speech throughout development, supports the view that strengthened top-down control may be one mechanism underlying musicians’ more precise auditory processing for both music and speech. Moreover, enhancements may already be evident following relatively few years of continuous music training in young children (Strait et al., 2013). Future research on this topic should clarify the relative dependence of learning on bottom-up and top-down processes during and after sensitive periods and the extent to which this balance is impacted by training. This is an exciting new field of research that may lead to new training methods geared towards optimizing learning across the lifespan.

## Conflict of interest statement

The authors declare that the research was conducted in the absence of any commercial or financial relationships that could be construed as a potential conflict of interest.

## Footnotes

^1^It is, however, important to note that these studies do not specify if the duration of musical training was the same for those who began music training before and after age seven. This means that the total number of years of musical training may also be important to maCC development, as well as age-of-onset of music training.

^2^Monaghan et al. (1998) criticized Pantev et al. (1998) because of the statistical techniques used (i.e., using two-tailed instead of one-tailed tests) and the correlational nature of the data without controlling for genetic or environmental effects. Subsequent research has supported Pantev et al.’s (1998) interpretation (e.g., see Shahin et al., 2004).

^3^Research into auditory scene analysis (i.e., how we form a meaningful auditory percept from multiple incoming auditory signals), also points to a facilitative developmental role of attention in audition and suggests a potential for music training for reading/language rehabilitation. Sussman and Steinschneider (2009) compared the amount of frequency separation that children and adults require to perceive two separate sound streams in active and passive listening conditions (i.e., with or without attention). In contrast to adults, who displayed similar ERP indices of sound segregation in both conditions, children required much larger frequency separation in passive compared to active listening conditions. This suggests that attention plays an important developmental role in shaping the neural networks underlying sound pattern organization used in passive listening conditions. Such findings may be particularly pertinent for children with language and/or reading impairment, who have been shown to have difficulty with sound segregation (i.e., require larger temporal or spectral differences to perceive segregated streams; Sutter et al., 2000) and who, according to some theories, suffer from impaired attention control (Petkov et al., 2005). To the extent that music training is associated with enhanced sound segregation (Zendel and Alain, 2009), music training may provide an important vehicle for reading/language rehabilitation.

^4^Not all studies report superior visual attention skills in musicians relative to non-musicians. For example, Strait et al. (2013) report a significant difference between musicians and non-musicians in auditory, but not visual, attention, as assessed by reaction time to particular (visual or auditory) target stimuli. In contrast, Moreno et al. (2011a) found an advantage of musical training on visual attention using ERP indices of response inhibition using a go/no-go procedure. Differences between studies may be due to the use of behavioural versus electrophysiological measures, assessing attention to a target, versus response inhibition to a distracter as well as the operationalization of what attention is.
